# Prepared for what? addressing the disaster readiness gap beyond preparedness for survival

**DOI:** 10.1186/s12889-015-2440-8

**Published:** 2015-11-17

**Authors:** Monica E. Gowan, Jeff A. Sloan, Ray C. Kirk

**Affiliations:** Independent Consultant, Seattle, WA USA; Mayo Clinic and School of Health Sciences, Rochester, Minnesota USA; College of Education, Health and Human Development, University of Canterbury, Christchurch, New Zealand

**Keywords:** Disaster resilience, Evacuation, Hazards, Health promotion, Health outcomes, Natural disasters, Preparedness, Prevention, Risk perception, Self-management

## Abstract

**Background:**

Conventional disaster preparedness messaging focuses largely on promoting survival actions and communications planning for the immediate post-disaster period. While such preparedness is vital, we have long-observed a gap in preventive medicine and disaster planning for building personal resilience – *preventatively* – to persevere through prolonged recovery timeframes. There are many helpful attitudes and behaviors that people can develop to increase their readiness and capacity for drastic life changes, encompassing not only health-protective preparedness actions but health-promoting attitudes for “minding the risk” and “practicing resilience” as well. For instance, quality of life assessments and well-being interventions are widely-known for the clinically significant improvements they can produce in patient-reported outcomes. Similarly, health promotion interventions are implemented preventatively when a risk is identified yet a disease is not present, and can provide health benefits throughout people’s lives, regardless of the type of adversities they eventually encounter (medical, environmental, or other).

**Discussion:**

We argue there is an overlooked opportunity to leverage well-being theories and methods from clinical settings and public health practice for the purpose of preventatively boosting disaster readiness and bolstering capacity for long-term resilience. We also highlight our previously-published research indicating a role for integrating personal meaning into preparedness messages. This is an opportune time for applying well-being concepts and practices as tools for developing disaster readiness, as risk awareness grows through real-time tracking of hazardous events via social media. For example, two sudden-onset disasters occurred within ten days of each other in 2014 and caught worldwide attention for their extreme hazards, despite dramatic differences in scale. The 22 March 2014 landslide tragedy in Washington State, USA, and the 1 April 2014 Chilean earthquake and Pacific-wide tsunami alerts brought home how persistently vulnerable we all are, and how developing intrinsic personal readiness for scientifically-known risks before disaster unfolds is essential policy.

**Summary:**

Gap programming that addresses personal readiness challenges in prevention timeframes could save lives and costs. We contend that bridging this readiness gap will prevent situations where people, communities, and systems survive the initial impact, but their resilience trajectories are vulnerable to the challenges of long-haul recovery.

## Background

Disaster preparedness messaging typically targets the most vital needs that arise during an extreme event and promotes resilience for the immediate post-disaster period. There certainly is a need for continued capacity-building to save lives, treat trauma, and to prepare people to be on their own during service delivery interruptions. Further, there is a well-established body of research and clinical practice on secondary and tertiary treatment (for morbid and co-morbid conditions, including Post Traumatic Stress Disorder) that has clearly led to improved outcomes for countless people and will continue to be extremely important. However, we have long observed a gap in preventive medicine and disaster planning for building adaptive capacity [1] in the preparedness phase, especially attitudes and behaviors that can help people persevere through prolonged recovery timeframes.

Indeed, those of us in the field, and those with disaster experience, are especially aware of what a long haul recovery can be—sometimes up to a decade or longer. A predominant focus on disaster’s onset and immediate aftermath can thus create a conundrum, namely: *what is it, exactly, that we are preparing for?* Is it solely to survive, maintain services, and manage livelihoods with minimal disruption? [2]. Or is it to transcend shattered expectations and profound uncertainties as well? Survivorship presents a new reality, along with potentially unanticipated challenges that can inhibit resilient recovery. For example, insurance risk burdens are increasingly transferred to individuals [3]. Other situational stressors may be outside of one’s control, such as displacement or even social pressures to “bounce back” or return to “normal.” Cultivating the ability to be risk-aware, accepting of irreversible change, and capable of exercising human agency to select adaptive attitudes and behaviors can lead to personal resilience as a process and outcome. This is the core purpose of health promotion, enabling all people to increase control over and to improve their health [4].

## Discussion

In 2014, two dramatic geophysical events occurred within ten days of each other, the 22 March 2014 Washington State, USA, landslide [5], and the 1 April 2014 magnitude 8.2 Chilean earthquake [6], focusing worldwide public attention in real-time via social media on the capriciousness of natural hazards.

**Fig. 1:**
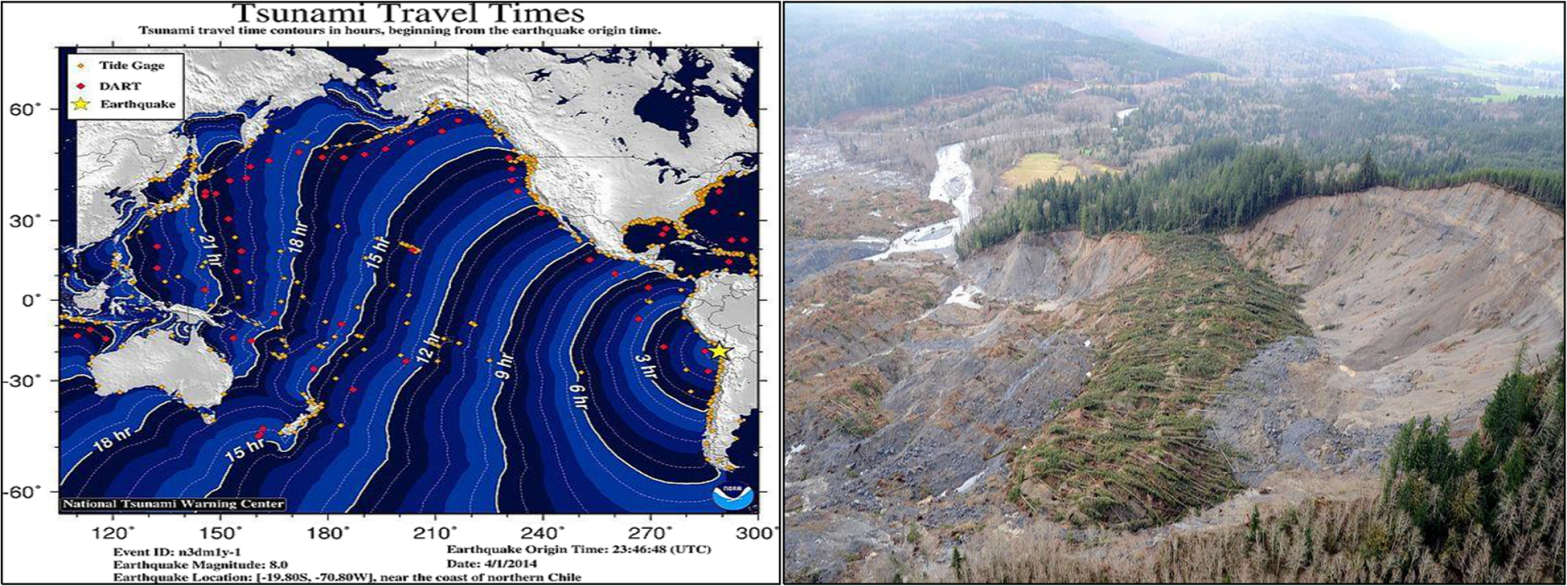
The 1 April 2014 Iquique, Chile earthquake and tsunami warning (*left*) and the 22 March 2014 landslide (*right*) across the North Fork Stillaguamish River valley near Oso, Washington, USA. These events had vastly different scales and hazard processes; however both required immediate evacuation and produced indiscrimnate effects. They also have long-range implications for international risk policy. *Image attribution*: Tsunami travel times map, National Tsunami Warning Center http://ntwc.arh.noaa.gov/previous.events/?p=04-01-14: Oso Landslide, Defense Video & Imagery Distribution System https://www.dvidshub.net/image/1209685/oso-mudslide#.VkTu-PkrKUl (public domain)

In Washington’s “Oso Landslide” (Fig. 1), a saturated hillslope collapsed in an area of previously-known landslide activity [7]; muddy debris swiftly buried an entire rural neighborhood of 49 homes and 43 people were lost. This unusually mobile slide [8] further dammed a river, caused flooding, spawned a mandatory downstream evacuation, and closed road access to the upstream communities. In Chile’s “Iquique Earthquake,” only 6 people perished, but nearly 1 million were evacuated along coastal Chile and Peru, experiencing extreme circumstances, personal distress, and for thousands, prolonged displacement [9]. Tsunami warnings were issued for the Latin American Pacific coastline (Fig. 1). Hawaii was under a tsunami advisory for over 13 h. Japan recorded 60 cm-high wave effects 2 days later [6].

### Minding the risk

These two events accentuated several readiness gaps and highlighted the following realities:

- No person, place or thing is invulnerable to disaster, simply by virtue of being in the wrong place at the wrong time.
- Disaster preparedness is more than preparing to survive an event; it is also building capacities in people to adapt—attitudinally and behaviorally—to future catastrophic transformations in their landscape, built environment, and everyday life.
- Disasters frequently and suddenly displace people from home and workplaces, sometimes never to return; this can profoundly change lives and livelihoods, especially for socially vulnerable populations;
- Personal costs and timelines for recovery are often ‘unthinkable’ or difficult to anticipate, introducing a high degree of uncertainty.
- Ruination does not require a large-scale event (e.g., the near-complete burial of the small rural neighborhood by the Oso Landslide), and less-than expected consequences do not preclude significant life disruptions (e.g., low rates of mortality yet wide-scale displacement in Chile).

Natural disasters will keep happening. On 16 September 2015, the magnitude 8.3 “Illapel Earthquake” struck Chile, killing at least 15 [10]. Once again, evacuation affected about 1 million people and Pacific-wide tsunami warnings were issued.

Notwithstanding significant advances in tsunami warning systems over the last decade, continued improvements in seismic safety codes, and better survival and evacuation planning, there is still ample room for improving how people learn and think about disaster risk, uncertainty, and resilience, and what they do to reduce their vulnerability. The persistence of the disaster experience and perceptual errors of risk lead us to reason that health promotion interventions are imperative for: 1) exorcising people’s commonly-held erroneous beliefs that they are less likely than others to experience misfortune [11] (e.g., “false optimism”); 2) solidifying commitment to readiness for low-probability high-consequence events; and 3) developing personal resilience that can transcend the event timeframe.

Fortuitously, access to risk information has grown dramatically through social media and the Internet over the last ten years and the public appetite for relevant, timely natural hazards information is increasingly robust. Research has also emerged indicating that personal preferences play a strong role in precautionary behavior leading to long-term hazard adjustments [11, 12]. These trends support an argument that thinking about risk must be personalized and that health promotion is a valuable approach for building personal resilience.

### An example from the evidence base: New Zealand evacuation preparedness

Baseline quantitative data provide much-needed evidence for developing indicators and offer support for promoting resilience preventatively [13]. We refer the reader to our previous research, a survey of the general adult population (*n* = 695) in Wellington, New Zealand, on their evacuation preparedness for earthquake and tsunami disaster [14]. Inferential analyses indicated significant positive associations among health-related quality of life and well-being; the strongest correlations with preparedness actions were evident with emotional and spiritual well-being. Overall health and well-being explained 5–7 % of the variance in evacuation preparedness. Spiritual well-being was a statistically significant unique predictor of evacuation preparedness. Preparedness was independent of gender and increased only slightly with age.

These results indicate a need for policies and practices that promote engagement in *personally meaningful* health-protective actions in advance of disaster. Taking this stance also offers an opportunity: leveraging people’s individual strengths and resources, while helping them learn and think about how to live with risk and uncertainty, may empower them to develop lifelong adaptive capacities. Further, evidence suggests that preparing for an uncertainty, even one that does not eventually transpire, can produce substantial and meaningful outcomes [15].

### Preventive practice and promoting readiness

We can go beyond the scope of current practices for survival and economic recovery to a broader horizon of readiness by integrating the properties of human agency—intentionality, forethought, self-regulation, and self-reflection—into disaster planning [16]. This will require personal risk awareness *and* clear pathways towards personally meaningful choices for individual well-being and readiness. When people are empowered to use their resources to confront natural forces and external challenges and move forward positively, stronger foundations for disaster resilient societies can result. A real-time cultural example emerged during the 2015 Illapel Earthquake: the Chilean approach of promoting calm (“tranquilo”) during intense seismic shaking [17]. This is not to say that failing to take protective action is advised, but that Chileans are aware they have a choice about how to respond and that remaining calm is recognized as beneficial and adaptive.

A window of opportunity is present, now further reinforced by global coverage of the Illapel Earthquake, for health professionals to intercede with comprehensive readiness programs. Moreover, aftereffects persist for survivors of the Iquique Earthquake, Oso Landslide, and other Pacific Rim disasters, such as the ongoing Canterbury Earthquake Sequence in New Zealand [18]. People become particularly attuned to risks and engage in self-protective actions after profound or recent disaster experiences [19]. We add our voice to those who call for addressing *how effectively are we preparing?*; *why don’t we prepare adequately?*; *what actions are best to take* and *what is the most important message?* [20–25].

### Practicing resilience: evidence based recommendations

Specifically, platforms that include the following aspects can address the issues above and lead to readiness and resilience as day-to-day processes and post-disaster outcomes:

- First, *promote health equity* in readiness campaigns. Prepare all people to be affected by disaster and displaced, as was done in Chile. Develop broad-brush interventions with consistent messages that are flexible enough to meet the complex and deeply personal needs of everyone.
- Second, *continuously engage all people in multi-faceted survival planning*. Build knowledge of how services and resources will be impacted and assist people in developing solutions for their physical needs through *functional needs planning* (e.g., for power, water, sanitation, food, transportation, medical needs, home and workplace safety). Involve people in *survival-and-revival evacuation planning* – assembling important documentation and getaway kits; planning escape routes and meeting places; participating in simulations, drills, responding to warning systems; and considering a place of refuge. Promote *multi-channel personal communications planning* using redundant strategies (including social media) for system failures or delays; maintaining charged personal electronic devices and recharging options during power failure; designating and reaching a remote communications relay person; and accessing relief services for emergency communications and other support. In the Washington example, uncertainties about survivorship were sadly endured by loved ones, responders (working at great personal risk), emergency managers, and a gripped public; anxieties can be lessened everywhere with stronger readiness messages for making plans for post-disaster rendezvous points and relaying messages.
- Third, vigorously engage people in constructing their personal health narrative and health identity: *What makes me feel healthy? What is required for me to be healthy, ready, and resilient? What will most help me?* A reasonable sense of personal control can powerfully motivate change, amplify coping, and lead to autonomy and self-determination, all important factors for resiliency. Disaster wellness planning can be advanced by cross-training preventive medicine and health specialists with emergency management professionals in the basics of health literacy and risk reduction and resilience strategies.
- Fourth, *promote mental and emotional preparedness as vital signs* of disaster readiness. Risk awareness and acceptance of grief and shock as natural consequences of disaster are essential; arming people with positive coping mechanisms is important for short-term safety and long-term outcomes. Build awareness that resilience trajectories are expressed variably between and within people over time; acceptance and compassion for others (and oneself) is also vital for resilient communities. Integrating mindfulness messages into national campaigns, community partnerships, and volunteer responder initiatives is one option. At the population level, some likely benefits of greater personal presence include less panic and fault-finding before the cause or consequences of disaster emerge, which unfortunately transpired within hours of the Washington landslide [7, 8].
- Fifth, promote attitudes and behaviors of “*readiness*” through interventions and education. Identify options for limiting personal risks and building stress resistance through “readiness challenges”: 1) Am I ready in thought (“*I know disaster can happen to me*”); 2) Am I ready in belief? (“*Disaster can be managed; I know my situation and how to access my strengths and resources – I know what sustains my physical, mental, emotional, social, spiritual, and overall well-being*”); and 3) Am I ready in action? (“*I am building and integrating my resources and capacities to act, adapt and flow within my own dynamic situation”*).

Finally, support people in exploring, *what will I do once I have survived? What will be most personally meaningful and useful?* Our data set provides evidence for prioritizing meaningfulness within pre-event resilience interventions [14]. Creating space for reflection to process and make meaning of risk can heighten awareness of what is personally important and thus prudent for one’s life. Meaningfulness, whether cultivated pre- or post-event, can serve as a tribute to hardships encountered throughout life, enrich the present moment and future potential [26], and move people and communities beyond the readiness gap to disaster resilience.

## Conclusion

In conclusion, gap programming that addresses disaster readiness outside the dominant paradigm of physically preparing for survival and preventatively builds intrinsic resilience for well beyond disaster’s initial impact could save lives and costs. We contend that bridging this readiness gap will prevent situations where people, communities, and systems survive the event but their resilience trajectories are vulnerable to the challenges of long-haul recovery.
